# How to Cope with Coping in Adult Post-Hemorrhagic Patients Undergoing Neurorehabilitation: A Scoping Review

**DOI:** 10.3390/jcm14145121

**Published:** 2025-07-18

**Authors:** Davide Cardile, Irene Cappadona, Erika Patti, Aurora Ansaldo, Rosaria De Luca, Francesco Corallo, Maria Pagano, Anna Anselmo, Angelo Quartarone, Rocco Salvatore Calabrò

**Affiliations:** 1 IRCCS Centro Neurolesi “Bonino-Pulejo”, 98124 Messina, Italy; davide.cardile@irccsme.it (D.C.);; 2 Department of Clinical and Experimental Medicine, University of Messina, 98122 Messina, Italy; 3 Department of Biomedical, Dental Sciences and Morphological and Functional Images, University of Messina, 98122 Messina, Italy

**Keywords:** intracerebral hemorrhage, acquired brain injury, coping strategies, functional recovery, neurorehabilitation

## Abstract

**Background/Objectives:** Cerebral hemorrhage (CH) has physical, cognitive, and emotional consequences. Recovery requires a complex rehabilitation process in which coping strategies play a fundamental role in supporting psychological adaptation. The aim of this study is to investigate and understand the extent and manner in which coping strategies have been assessed in the CH population within the scientific literature. **Methods:** Studies were identified through searches in the PubMed, Scopus, and Embase databases. Eight studies published between 2014 and 2024 were selected. **Results:** The most frequently adopted coping strategies include task-oriented coping, avoidance, emotion-focused coping, acceptance, planning, and emotional support. Task-oriented strategies and acceptance are associated with better psychological outcomes. Conversely, avoidant and emotion-focused strategies correlate with higher levels of anxiety, depression, and poorer adaptation. Resilience and social participation emerge as protective factors. Finally, Action/Distraction is associated with a better quality of life, while Trivialization/Resignation is linked to lower levels. **Conclusions:** Coping seems to represent a modifiable, patient-centered lever that can mitigate the psychosocial sequelae of intracranial hemorrhage when assessed systematically and addressed through tailored rehabilitation programs. Our findings lay the groundwork for evidence-based, coping-focused interventions and highlight critical avenues for future longitudinal and mechanistic research.

## 1. Introduction

Intracranial hemorrhage (ICH) is defined as any pathological extravasation of blood within the cranial vault and embraces spontaneous intraparenchymal (intracerebral) hemorrhage, aneurysmal or non-aneurysmal subarachnoid hemorrhage (SAH), and traumatic hemorrhagic lesions that may be epi- or subdural, subarachnoid or intraparenchymal in location [1,2,3]. Collectively, these events account for ~40 % of stroke-related mortality and remain a major contributor to neurological disability worldwide [4,5].

Despite this shared endpoint, the etiological and pathophysiological mechanisms precipitating bleeding differ substantially: hypertensive microangiopathy or cerebral amyloid angiopathy in spontaneous ICH, aneurysmal rupture and early brain injury cascades in SAH, and linear or rotational acceleration forces in traumatic brain injury (TBI) [6,7,8]. Secondary injury processes—inflammation, excitotoxicity, oxidative stress and perilesional edema—evolve over days to weeks and critically influence long-term functional trajectories. Generally acute symptomatology includes sudden headaches, rapid loss of consciousness within seconds or minutes, nausea, vomiting, delirium or mental confusion, seizures, and hemiparesis [9,10]. However, cognitive and emotional morbidity constitutes one of the most pervasive yet least visible legacies of ICH. Prospective cohort studies demonstrate that 50–80 % of survivors exhibit deficits in at least one cognitive domain six months post-event, with executive dysfunction, slowed processing speed and impaired working memory being most prevalent [11,12,13]. Lesion topography modulates the pattern of impairment: deep hemispheric bleeds and diffuse traumatic contusions preferentially disrupt frontostriatal circuits, whereas aneurysmal SAH often compromises mesial temporal and limbic networks, producing episodic memory deficits, alexithymia and emotional dysregulation [14,15]. Diffuse axonal injury, cerebral vasospasm, hydrocephalus and delayed cerebral ischemia further exacerbate these deficits.

The neuropsychiatric burden is equally striking. Systematic reviews estimate that up to two-thirds of patients develop major depressive symptomatology within the first year, and one-third fulfil criteria for anxiety disorders or post-traumatic stress, regardless of hemorrhage subtype [16,17,18]. Fatigue, apathy and emotional lability—collectively termed the *affective motivational syndrome*—have been linked to monoaminergic pathway disruption and frontolimbic disconnection [19]. Sleep–wake disturbances, chronic pain and endocrine dysregulation act as synergistic amplifiers of psychological distress [20,21]. Importantly, these “invisible” sequelae predict poorer community reintegration, heightened caregiver burden and diminished health-related quality of life, even after motor disability is accounted for [22,23,24].

Optimal management should commence in a specialized hospital environment—typically a neurocritical-care or stroke-unit setting—where coordinated input from neurologists, neurosurgeons, neurointensivists, emergency physicians, internists, rehabilitation physicians, neuropsychologists, speech language pathologists, occupational and physical therapists, and specialized nursing staff can be delivered in a time-critical and protocol-driven fashion. Evidence from implementation trials and population registries demonstrates that such multidisciplinary models of care are associated with lower early mortality, reduced medical complications, and superior 6-month functional independence when compared with treatment in general medical wards [6,7,8]. Within this framework, early and continuous neurorehabilitation is initiated to foster recovery and facilitate adaptation to residual disability. Rehabilitation programs combine task-oriented motor training, cognitive remediation, psychosocial education, and caregiver counseling, all tailored to lesion characteristics, comorbidity profile and premorbid functioning [12,13]. Importantly, individuals with ICH frequently display pronounced emotional fragility during the subacute phase, typified by sadness, fear, irritability and frustration that can erode engagement with cognitive-behavioral therapies and impede long-term adaptation [14,15]. Systematic mood and anxiety screening, timely psychopharmacological or psychotherapeutic intervention, and caregiver-inclusive counseling are therefore integral to comprehensive care.

Within this context, coping strategies—the continuously evolving cognitive and behavioral efforts deployed to handle demands appraised as taxing or exceeding one’s resources—emerge as pivotal drivers of engagement in neurorehabilitation and long-term psychosocial adjustment [25,26]. Grounded in the transactional model of stress proposed by Lazarus and Folkman [15], coping responses after ICH can be broadly categorized into (i) emotion-focused coping, aimed at modulating negative affect; (ii) problem-focused coping, which seeks to modify the stressor itself; (iii) active coping, characterized by direct, solution-oriented actions; (iv) avoidant coping, typified by denial, distraction or behavioral disengagement; (v) accommodative coping, wherein personal goals are flexibly adapted to emergent limitations; and (vi) assimilative coping, which endeavors to reshape the environment to fit premorbid aspirations [27].

The converging literature demonstrates that a predominant reliance on emotion-focused or avoidant strategies is associated with poorer emotional adjustment, diminished self-esteem and elevated depression, anxiety, apathy and denial, whereas preferential use of problem-centered, active or accommodative approaches predicts superior health-related quality of life, higher participation indices and reduced caregiver burden [28,29,30]. Importantly, the cognitive sequelae of ICH—particularly deficits in executive functioning, attentional control and working memory—can constrain goal-directed problem solving and bias survivors towards maladaptive coping modes, such as rumination or withdrawal [31,32].

Accordingly, systematic and early appraisal of coping styles using validated instruments (e.g., Coping Inventory for Stressful Situations, COPENVI) serves a dual purpose: (1) it identifies individuals at heightened risk of chronic emotional distress and social isolation, and (2) it informs the tailoring of rehabilitation interventions to reinforce constructive coping through metacognitive strategy training, problem-solving therapy, motivational interviewing or acceptance and commitment techniques [33,34]. Emerging trials indicate that timely optimization of coping capacity can attenuate the incidence of secondary mood disorders, enhance adherence to high-intensity rehabilitation schedules and ultimately foster more favorable trajectories of community reintegration and life satisfaction after ICH [35].

The present systematic review therefore aims to investigate and understand the extent and manner in which coping strategies have been assessed in these populations in the scientific literature.

## 2. Materials and Methods

### 2.1. Search Strategy

**Figure 1:**
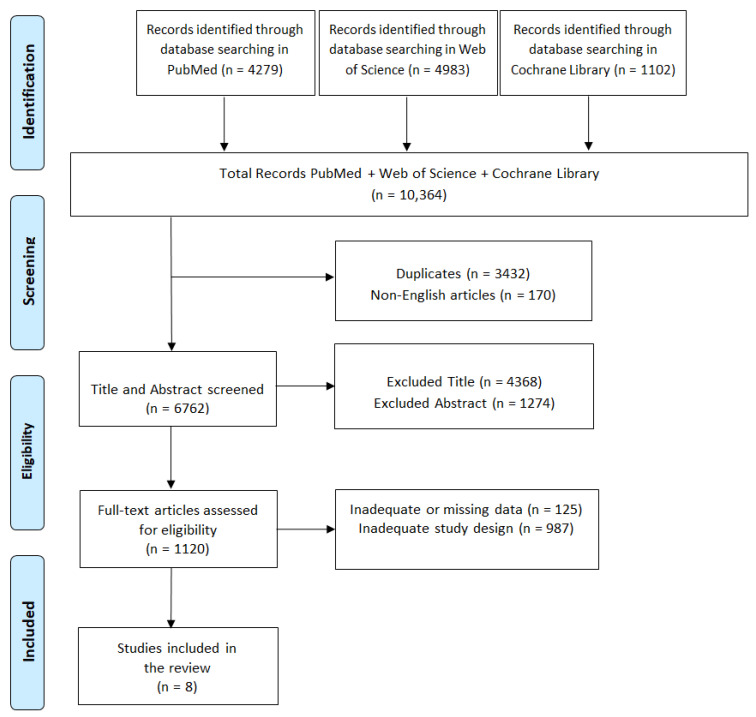
PRISMA flow-chart of the current review.

A review of currently published studies was conducted in line with the Preferred Reporting Items for Systematic Reviews and Meta-Analysis (PRISMA 2020) guidelines [36] (Figure 1). Relevant articles were searched before April 20, 2024, on English electronic databases, including PubMed, Web of Science, and Cochrane Library, and were considered only if published between 2014 and 2024. The specific search strategy used for all databases is: ((coping strategies [Title/Abstract]) AND (cerebral hemorrhage [Title/Abstract])) OR (neurorehabilitation [Title/Abstract]). ((Intracranial hemorrhage [Title/Abstract]) OR (intraparenchymal hemorrhage [Title/Abstract]) OR (intracerebral hemorrhage [Title/Abstract]) OR (subarachnoid hemorrhage [Title/Abstract]) OR (traumatic hemorrhagic [Title/Abstract]) AND (neurorehabilitation [Title/Abstract])) AND (coping strategies [Title/Abstract]) OR (neurorehabilitation [Title/Abstract]).

### 2.2. Study Selection

To minimize bias and ensure a rigorous selection process, two authors (D.C. and I.C.) independently reviewed and extracted data from the studies. Any discrepancies were resolved through collaborative discussion, with consultation from a third author (F.C). This multi-step approach ensured that at least three researchers independently assessed each article. In cases of persistent disagreement, the final decision involved all authors.

### 2.3. Inclusion Criteria

A study was included if it described or investigated coping strategies in patients with ICH. Only articles written in English and published within the past 10 years were included in the review.

### 2.4. Exclusion Criteria

A study was excluded if there was a lack of data or information about the coping strategies in patients with ICH. Systematic, integrative or narrative reviews were also excluded, although their reference lists were reviewed and included if appropriate. All articles written in languages other than English were excluded.

### 2.5. Risk of Bias Within Individual Studies

**Figure 2:**
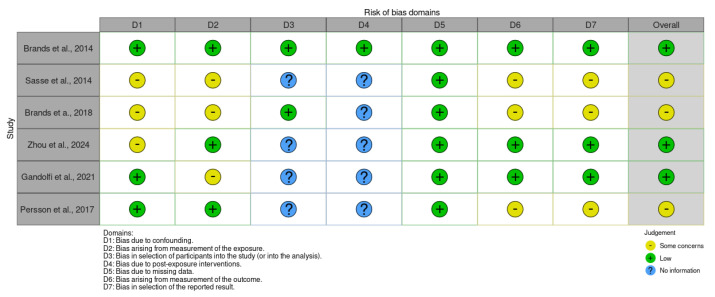
Risk of bias (ROBINS-E) of the included studies [38,39,40,41,42,43].

The risk of bias was assessed using the Cochrane tool for non-randomized controlled trials of exposures (ROBINS-E) [22] (Figure 1), which includes seven domains: (i) bias due to confounding, (ii) bias from exposure measurement, (iii) bias in the selection of study participants (or in the analysis), (iv) bias due to post-exposure interventions, (v) bias due to missing data, (vi) bias from outcome measurement, and (vii) bias in the selection of the reported outcome. The risk of bias was also assessed using the Cochrane tool for randomized controlled trials of exposures (ROB2) [37] (Figure 2), which includes five domains: (i) bias due to the randomization process, (ii) bias due to deviations in the intended interventions, (iii) bias in missing outcome data, (iv) bias due to risk of bias in the measurement of post-exposure outcomes, and (v) bias due to risk of bias in the selection of the reported outcome.

## 3. Results

### 3.1. Synthesis of Evidence

In total, 9549 articles resulted from the initial electronic data search. A total of 3344 articles were removed after screening due to duplication. A total of 161 articles were excluded because they were not in English. A total of 4936 articles were removed based on title and abstract screening. Finally, 1101 articles were removed based on screening for inadequate study designs and untraceable articles. Eight research articles met the inclusion criteria (Figure 1).

### 3.2. Risk of Bias Results

The findings reported in Figure 2 emerged from the Cochrane tool for non-randomized controlled trials of exposures (ROBINS-E).

**Figure 3:**
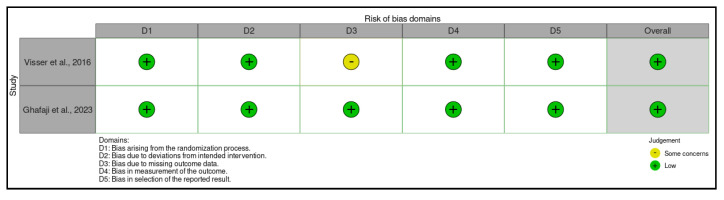
Risk of bias (ROB2) of the included randomized trials [44,45].

The latter shows that in the domain of bias due to confounding, three studies [39,40,41] raise some concerns, while three studies [38,42,43] show a low risk. In the domain of bias in exposure measurement, three studies [39,41,42] indicate some concerns, while three studies [38,40,43] show a low risk. In the domain of bias in participant selection, four studies [39,40,42,43] present a lack of information, while two studies [38,41] show a low risk. In the domain of bias due to post-exposure interventions, five studies [39,40,41,42] highlight a lack of information, and only one study [38] presents a low risk. In the domain of bias due to missing data, all studies [38,39,40,41,42,43] indicate a low risk. In the domain of bias in outcome measurement, three studies [39,41,43] identify some concerns, while three studies [38,40,42] show a low risk. In the domain of bias in the selection of the reported outcome, three studies [39,41,43] highlight some concerns, while three studies [38,40,42] show a low risk. Overall, three studies report an overall low risk of 60% [38,40,42], while three studies [39,41,43] indicate some concerns, accounting for 40%. The results from the Cochrane tool for randomized controlled trials of exposures (ROB2) are reported in Figure 3.

In the domains of bias due to the randomization process, bias due to deviations from intended interventions, bias due to the risk of bias in post-exposure outcome measurement, and bias due to the risk of bias in the selection of the reported outcome, all studies [44,45] show a low risk. In the domain of bias in missing outcome data, one study [44] raises some concerns, while one study [45] indicates a low risk. Overall, the studies show a low overall risk (100%).

### 3.3. Key Findings from Included Studies

All studies included in the review examined the topic of coping strategies in patients with ICH. See Table 1 for a detailed description of the studies and Table 2 for the psychometric tests used.

The number of studies published between 2014 and 2024 included in our research was seven. Within this sample, three studies were multicenter non-randomized cross-sectional studies [38,39,40], one study was a multicenter randomized controlled trial [44], one study was a non-randomized longitudinal cohort study [31], one study was a non-randomized single-center cross-sectional study [42], and, finally, one study was a randomized controlled cross-sectional study [45]. The number of patients included ranged from 50 [42] to 239 [40] (mean: 150). Although they may have differed in objective, all studies involved administered batteries and/or specific tests to generally or specifically assess coping strategies. Often, this evaluation was carried out in conjunction with other constructs. Specifically, it was examined alongside depression [38,39,41,44,45], anxiety [38,39,41,45], quality of life [39,42,44], and social aspects [40,42]. The tests used for the specific assessment of coping strategies were the CISS, AACQ, FQCI, COPE, the Brief-COPE, and the SCSQ. The CISS was used by three studies [38,41,44], while the other tests were employed only once.

The results obtained by the coping strategies assessment indicate that a lower flexibility leads to less reliance on task-oriented strategies [38,42,44]. In patients with higher resilience scores, social participation increased, and, consequently, the use of positive coping strategies rose [40]. Emotion-oriented coping correlates positively with anxiety and depression [38,39]. Brands et al. reported that the most commonly used styles are task-oriented coping, followed by avoidance- and emotion-oriented coping [41]. Ghafaji et al. [45] identified as most frequently employed strategies Acceptance, Emotional Support, Active Coping, and Planning. Acceptance appeared inversely correlated with fatigue, suggesting a beneficial effect. Patients with greater mental fatigue and emotional symptoms used more maladaptive avoidance strategies [41,45] and often exhibited lower QoL scores [39,44]. Coping strategies adopted also include support from family, society, employers and the use of technical equipment [43]. Action/Distraction and Trivialization/Resignation were identified as common coping factors after TBI. Specifically, the former appears to correlate positively with HRQoL, while the latter correlates negatively. Both factors are related to anxiety, depression, recovery, cognitive status, mood, and trauma severity [39].

## 4. Discussion

Coping comprises the set of mental, emotional, and behavioral strategies implemented by an individual to cope with and manage stressful, difficult, and traumatic situations [68]. These strategies can be functional, promoting psychological adaptation and well-being, or dysfunctional, exacerbating emotional distress or hindering the recovery process [28]. In patients with ICH, these dynamics assume a key role, because the psychological adjustment pathway requires the management of physical and emotional challenges. Given the importance of coping in the process of adapting to an illness, we considered it essential to analyze how the scientific literature has addressed this issue in relation to this clinical condition.

### 4.1. Most Common Coping Strategies in Patients with Cerebral Hemorrhage

The results of our study show that the coping strategies most frequently adopted by patients with ICH include task-oriented coping, avoidance, emotion-oriented coping [41], acceptance, emotional support, active coping, and planning [45]. These findings are consistent with the scientific literature, which indicates that patients who use active, problem-focused coping strategies after a traumatic brain injury tend to exhibit greater resilience, a higher sense of self-efficacy, and fewer symptoms. Directly addressing difficulties, for example, through the search for concrete solutions, is often associated with more adaptive functioning [69]. However, several studies highlight that in patients with brain injury and executive dysfunctions, the use of problem-focused strategies is less frequent, likely due to cognitive limitations that hinder effective problem-solving [70,71,72,73]. In such cases, emotion-focused strategies are more commonly employed.

In the early stages after a severe traumatic brain injury, or in the presence of significant cognitive deficits and ICH, avoidance or emotion-based strategies are more frequently observed [74,75]. Awareness of one’s own condition plays a crucial role: individuals with greater awareness tend to use task-oriented strategies, while those with limited awareness tend to rely more on avoidance. Prolonged use of passive or avoidant strategies, especially in the chronic phase of brain injury and trauma, has been associated with worsening mood and quality of life. In contrast, acceptance has proven to be an adaptive strategy, promoting adjustment to the illness, reducing fatigue, and contributing positively to quality of life [74]. Our results also support this evidence, as acceptance was found to be inversely correlated with fatigue, suggesting a beneficial effect. Patients reporting greater mental fatigue and emotional symptoms were more likely to adopt maladaptive avoidant strategies [41,45], while also showing lower scores in quality of life [39,44].

### 4.2. Emotional Coping and Its Impact on Anxiety and Depression

Our study reveals that emotion-focused coping is positively related to anxiety and depression in patients with brain hemorrhage [38,39]. This finding suggests that, in the context of severe neurological disease, the predominant use of emotion-focused coping strategies may be associated with a deterioration in psychological well-being. Consistent with previous studies [76,77,78], it has been observed that brain injury patients and their family members often experience symptoms of anxiety and depression. These psychological conditions in patients are often a response to the functional limitations imposed by the disease, the loss of personal autonomy, and the profound changes that brain injury brings to daily life. Depression and anxiety also have a significant impact on social and cognitive functioning. In particular, they can impair the ability to maintain social relationships, organize daily activities, and complete tasks important to the patient. In addition, they can hinder even simple activities that require concentration and mental clarity [79]. Approaches such as cognitive-behavioral therapy, psychoeducation, and integrated psychological support within neurological rehabilitation programs, targeted at both patients and their families, have proven effective in reducing symptoms of anxiety and depression and in improving quality of life [80,81,82,83]. In this context, cognitive difficulties such as reduced mental flexibility also play a central role in determining a patient’s ability to cope with everyday challenges.

### 4.3. Cognitive Flexibility and Problem-Focused Coping

Our research highlights that lower cognitive flexibility is associated with reduced use of task-oriented coping strategies in patients with ICH [38,42,44]. These findings are consistent with evidence from studies on stroke and chronic illnesses, which emphasize the crucial role of psychological flexibility in adaptive functioning. In this context, therapeutic interventions aimed at enhancing psychological flexibility, such as Acceptance and Commitment Therapy (ACT), have proven effective in supporting the management of chronic illnesses and in reducing related emotional distress [84]. Even in post-stroke rehabilitation, acceptance of one’s condition and its consequences has emerged as a key factor in the adjustment process [85]. A preliminary study [86] showed promising results with a brief group-based ACT intervention: participating patients reported improvements in coping strategies, suggesting that this approach may be a valuable complement to traditional rehabilitation programs. Overall, these findings underscore how developing greater flexibility not only facilitates adaptation to illness but also strengthens relational skills and encourages active engagement in social life. This perspective aligns with the concept of resilience, understood as a key resource for promoting meaningful and sustainable participation in the post-rehabilitation context.

### 4.4. Resilience as a Protective Factor and Facilitator of Social Participation

The results of our review show that patients with higher resilience scores also demonstrate increased social participation, along with a greater use of positive coping strategies [40]. These findings are consistent with previous studies [87,88], which emphasize that the way individuals cope with stressful events influences their social behavior. In patients with ICH, the ability to maintain or rebuild meaningful social relationships can play a decisive role in both emotional and functional recovery. Socialization, in fact, supports better stress management: interacting with others helps individuals develop new strategies to deal with everyday challenges. Activities such as mutual listening or collaborative problem-solving can strengthen a sense of connection and support, creating a positive cycle that reinforces itself over time [89]. However, it is essential to consider not just the quantity, but also the quality of social interactions. High levels of social participation do not always equate to well-being: when relationships are superficial, lack empathy, or are not genuinely supportive, their impact on coping may be limited, or even counterproductive [89,90].

### 4.5. The Impact of Active and Passive Coping on Quality of Life

Finally, the study by Sasse et al. [39] found that the use of the Action/Distraction strategy—consisting of staying active and using functional distractions to cope with difficulties—is associated with better health-related quality of life (HRQoL) in patients with ICH. In contrast, coping strategies such as Trivialization/Resignation, characterized by passive minimization of the problem or a defeatist attitude, are correlated with lower quality of life. These results are supported by other studies. For example, [91] observed that the adoption of active strategies—including engagement in recreational activities and social participation—is associated with better psychosocial outcomes and improved quality of life in patients with traumatic brain injury. Similarly, several studies [28,92] have reported that active coping is linked to lower levels of depression and anxiety, as well as better overall functioning, in patients with aneurysmal subarachnoid hemorrhage and acquired brain injury. The beneficial effects of these strategies can be attributed to the fact that active coping promotes a sense of control and self-efficacy—two psychological factors that are fundamental for adapting to chronic conditions. Distracting oneself through meaningful activities or continuing to pursue personal goals can help maintain daily routines, reinforce personal identity, and reduce rumination, which is often associated with depressive and anxious states [91]. Conversely, passive strategies, such as resignation or trivialization of the problem, tend to hinder the adjustment process, fostering attitudes of passivity and social isolation. These coping styles are frequently associated with feelings of helplessness and a gradual worsening of mood. Various studies [93,94,95] have demonstrated that prolonged use of avoidant or passive strategies is linked to deteriorating psychological well-being, increased depressive symptoms, and reduced quality of life during the chronic phase post-injury and in chronic illness contexts.

### 4.6. Psychological Interventions for Improving Coping and Clinical Outcomes

The inclusion of evidence-based therapies, such as CBT (Cognitive Behavioral Therapy), EMDR, ACT, and family therapy, can represent a crucial strategy to enhance coping styles and clinical outcomes in the course following stroke and cerebral hemorrhage.

A recent meta-analysis shows that CBT significantly reduces anxiety and depression up to three months after treatment in post-stroke patients [96]. Moreover, another study highlights benefits on quality of life and improvement in self-efficacy [97].

Regarding PTSD, a clinical case of EMDR delivered via telemedicine post-stroke achieved symptom remission within a few sessions and reduced anxiety and depression [98], while a multiple-case study involving patients with neurological injuries found complete PTSD resolution in four cases, maintained at three months post-intervention [99]. This suggests that EMDR may promote more adaptive coping by facilitating early emotional processing and interrupting dysfunctional avoidance processes [100]. Furthermore, supportive therapy administered early post-stroke has demonstrated significant reduction of PTSD symptoms compared to standard health education [97].

Interventions focusing on coping, resilience, and family support show positive effects not only on the individual patient but also on caregiver well-being: caregiver self-efficacy at two months predicts lower anxiety and depression in patients at twelve months [101]. Couple-based interventions, designed to support dialogue and shared stress management, have shown improvements in individual and dyadic coping, with increased quality of life and reduced depressive symptoms [101,102].

Finally, studies on ACT have demonstrated that brief group interventions delivered by nurses in hospital settings can reduce depressive symptoms, improve sleep quality, and increase hope, with effects sustained up to three months post-treatment [86]. Another study confirmed that ACT promotes better emotional regulation and reduces experiential avoidance in neurological patients, suggesting a lasting transformation of coping style from passive and avoidant to mindful and active [103].

In conclusion, integrating these interventions into post-stroke and cerebral hemorrhage care could represent a significant opportunity not only for managing psychological symptoms but also for improving coping styles and long-term functional and relational outcomes.

### 4.7. Limitations and Strengths

Our study presents several strengths. In particular, to the best of our knowledge, it represents the first review specifically focused on the topic of coping in patients with ICH, a field that has been scarcely explored in the scientific literature so far. Moreover, the review highlights how coping strategies are a fundamental element for the psychological well-being and quality of life of these patients. Deepening this aspect is therefore crucial to improve the quality of the multidisciplinary approach, enabling the design of specific and targeted interventions. These interventions are essential to effectively support the psychological adaptation of patients and promote their long-term well-being.

However, our research has some limitations. First of all, it included a limited number of studies, as only eight articles met the inclusion criteria. Furthermore, among the selected studies, only one adopted a longitudinal design, while the others were cross-sectional. This represents an additional methodological limitation, as cross-sectional designs do not allow capturing changes over time. Another critical issue concerns the lack of consensus on coping measurement tools. Only three of the studies included used the same instrument, namely the Coping Inventory for Stressful Situations (CISS), while the others employed different tools, making direct comparison of results difficult. Finally, the included studies did not exclusively concern cerebral hemorrhage, except for one study focused on aneurysmal subarachnoid hemorrhage; the other studies involved brain injuries where cerebral hemorrhage was also present.

### 4.8. Future Perspectives

In light of the results obtained, future research could explore how patients can learn effective and constructive coping strategies that are useful not only for managing immediate difficulties but also for accepting and living with the awareness of their diagnosis over the long term. Moreover, longitudinal studies would be essential to understand the evolution of coping over time and its impact on psychological and functional outcomes in the medium-to-long term. Another promising area concerns the use of telemedicine, which could offer new opportunities to monitor and support coping in a personalized, continuous, and remote manner. Finally, greater inclusion of caregivers’ perspectives would be desirable, in order to develop interventions that support not only the patient but also the family context as a whole.

### 4.9. Conclusions

Based on the identified research, we can confirm the importance of coping strategies in the management of acquired brain injuries, highlighting how cognitive flexibility, resilience, and self-awareness significantly impact patients’ quality of life. Task-oriented coping strategies, acceptance, and social support have proven particularly effective in promoting psychological adjustment, enhancing emotional well-being, and strengthening social participation. In contrast, the prolonged use of passive or avoidant strategies is associated with negative outcomes, such as increased fatigue, depression, and a decline in quality of life. These findings underscore the need for personalized interventions that encourage active and flexible coping strategies tailored to the patient’s residual cognitive abilities and level of self-awareness. In the rehabilitation process, it is essential to recognize the influence of these mechanisms on the management of everyday situations, as they directly affect the cognitive and emotional perception of the illness experience. This perception, in turn, forms the foundation for either functional or dysfunctional psychological and physical recovery outcomes. Therefore, rehabilitation interventions must include specific assessments and targeted support aimed at strengthening resilience and fostering adaptive coping strategies, calibrated to the individual needs and characteristics of each patient. In this context, the integration of evidence-based psychological interventions, such as CBT, EMDR, ACT, and family therapy, can be crucial. These approaches not only alleviate emotional distress but also promote the development of more adaptive coping styles, facilitate emotional processing, and enhance the overall recovery journey. Their inclusion in post-stroke and post-brain injury care programs is a key element in optimizing functional, emotional, and relational outcomes over the long term.

## Figures and Tables

**Table 1:** Characteristics of the studies.

Study	Aim	Type of Study	Population	Study Design/Intervention	Measures	Outcomes	Limitations
Brands et al., 2014 [38]	To examine the factorial structure, internal consistency and validity of the CISS in patients with ABI including ICH. Analyze the associations of the CISS with HADS and AACQ to assess its convergent and divergent validity.	Multicenter cross-sectional study.	109 patients	Rehabilitation physicians and neurologists recruited eligible and consenting patients. Patients completed the standardized tests by telephone after discharge or before the start of rehabilitation.	CISS; HADS; AACQ;	The three-factor structure is acceptable, internal consistency is good, discriminant validity is partial. Emotion-oriented coping is related to anxiety and depression. Task-oriented coping is associated with active goal seeking. Emotion-oriented coping is less used by flexible adjusters. CISS has acceptable-to-good psychometric properties.	Reduced sample for confirmatory factor analysis. Use of a specific instruction instead of the original CISS instruction.
Sasse et al., 2014 [39]	To examine the coping strategies adopted after a TBI, including the consequences of brain hemorrhage, and to analyze their associations with HRQoL.	Multicenter cross-sectional study.	141 patients	Participants were contacted by mail and telephone, providing written consent. The GOSE and TICS were collected by telephone interview, while the remaining questionnaires were mailed, completed, and returned by mail.	FQCI; SF-36; QOLIBRI: HADS; POMS; GOSE; TICS;	Two coping factors have been identified: Action/Distraction and Banalization/Reassignment. The former is positively associated with two domains of HRQoL, while the latter has a negative impact. Both factors are related to anxiety, depression, recovery, cognitive status, mood states, and severity of trauma.	Small sample size, varying time since injury. Exclusion of subjects with psychiatric disorders reducing generalizability. Use of the FKV-LIS instrument with some limitations in scale consistency.
Visser et al., 2016 [44]	To investigate whether problem-solving therapy (PST) increases coping strategies and HRQoL in patients with stroke, including subarachnoid hemorrhage.	Multicenter randomized controlled trial.	166 patients	Eligible patients were evaluated before (T0), after surgery (T1) and at 6 (T2) and 12 (T3) months after surgery. The control group received only outpatient rehabilitation, while the experimental group also received PST.	CISS; SPSI-R; SS-QOL; EQ-5D-5L; CES-D;	The experimental group showed significant improvement over the control group in task-oriented coping, avoidant coping and general HRQoL, but not in psychosocial HRQoL.	Absence of active intervention in the control group. Limited generalizability of results because the study took place in a post-stroke outpatient setting.
Persson et al., 2017 [43]	Exploring experiences of care and rehabilitation, consequences, and coping strategies adopted to deal with daily life six years after subarachnoid hemorrhage.	Cross-sectional study.	16 patients	Participants were contacted by phone and, upon giving their consent, an interview was scheduled. The interviews were conducted face-to-face.	Ad hoc questionnaire	Coping strategies adopted included support from family, society, employers, and the use of technical equipment.	The experiences reported by the participants are deeply influenced by the Swedish sociocultural context, which calls for caution when applying or generalizing the findings to other cultural settings.
Brands et al., 2018 [41]	To examine the use of coping styles and their relationship with emotional distress and HRQoL in ABI patients including ICH.	Longitudinal cohort study.	143 patients	The first evaluation was done after discharge (T0) the second evaluation was done after one year (T1). Questionnaires were mailed or administered in attendance.	CISS; LISAT-9; HADS;	Task-oriented coping was the most used, followed by avoidance and emotion-oriented coping. Lower CISS-E and HADS scores were associated with higher scores in LiSat-9. CISS-E had a direct effect on LiSat-9 and an indirect effect on HADS.	Sample composed mainly of men, with possible influence on coping strategies. Presence of patients at different stages of disease, with potential impact on coping strategies adopted.
Gandolfi et al., 2021 [42]	To analyze disability, quality of life, psychological distress, and psychological characteristics in post-stroke patients, comparing those with chronic pain and those without pain.	Cross-sectional study.	50 patients	After a screening telephone interview to verify inclusion and exclusion criteria, eligible patients completed paper questionnaires at the Neurorehabilitation Unit.	SIS 3.0; BPI; SCL-90-R; GSE; COPE; AAQ-II; MSPSS	Patients with chronic pain compared with those without pain showed greater disability, worse quality of life, greater psychological distress, less flexibility, reduced self-efficacy and limited use of problem-oriented coping strategies.	Small sample size. Lack of pre-registration of the study. Participants not fully representative of all stroke survivors as having sufficient cognitive level to complete questionnaires.
Ghafaji et al., 2023 [45]	To analyze the coping strategies of patients with post-aneurysmal subarachnoid hemorrhage fatigue and evaluate their association with fatigue severity and emotional symptoms in order to develop effective behavioral therapy.	Cross-sectional randomized controlled trial.	96 patients	Patients were recruited through telephone interviews and medical screening. They then independently completed questionnaires at home after receiving instructions from a neuropsychologist.	Brief-COPE; FSS; MFS; BDI-II; BAI;	The most frequently used coping strategies were Acceptance, Emotional Support, Active Coping, and Planning. Only Acceptance was inversely related to fatigue, suggesting a positive effect. Patients with greater mental fatigue and emotional symptoms used maladaptive avoidance strategies.	Limited generalizability due to strict inclusion criteria. Small sample size, limiting subgroup analysis. Lack of a fatigue-free control group post-aSAH.
Zhou et al., 2024 [40]	To analyze the relationship between resilience and social participation and evaluate the role of resilience as a mediator between coping strategies and social participation in post-stroke patients, including those with brain hemorrhage.	Multicenter cross-sectional study.	239 patients	Patients were recruited through advertisements at three neurorehabilitation centers. The researchers administered questionnaires, which the participants filled out independently. For the illiterate, the questions were read aloud.	USER-P; CD-RISC; SCSQ;	Higher resilience is associated with greater frequency of social participation, fewer restrictions in participation and greater satisfaction in participation. Higher resilience is related to positive coping strategies. Positive coping is associated with higher frequency of social participation.	Cross-sectional design prevents assessment of causality and directionality. Possible selection bias, as the sample is from three neural rehabilitation hospitals and mainly includes patients with severe functional impairment and limited social participation.

Legend: ABI: acquired brain injury; CISS: Coping Inventory for Stressful Situations; HADS: Hospital Anxiety and Depression Scale; TBI: head injury; HRQoL: health-related quality of life; AACQ: Assimilative/Assimilative Coping Questionnaire; FQCI: Freiburg questionnaire of coping with illness; SF-36: Short Form-36 Health Surve; QOLIBRI: Quality of life after brain injury; POMS; Profile of mood states; GOSE: Glasgow outcome scale extended; TICS: Cognitive Status Telephone Interview; PST: Problem-Solving Therapy; SPSI-R: Social Problem-Solving Inventory-Revised;SS-QOL: Stroke Specific Quality of Life Scale SS-QOL; CES-D: Center for Epidemiological Studies Depression; LISAT-9: Life-Satisfaction Questionnaire-9; SIS 3.0: Stroke Impact Scale; BPI: Brief Pain Inventory; SCL-90-R: Symptom Checklist-90; GSE: General Self-Efficacy; COPE: Coping Orientation to Problems Experienced; AAQ-II: Acceptance and Action Questionnaire—version 2; MSPSS: Multidimensional Scale of Perceived Social Support; Brief-COPE: Coping Orientation to Problems Experienced Inventory; FSS: Fatigue Severity Scale; MFS: Mental Fatigue Scale; BDI-II: Depression Inventory II edition: BAI: Beck’s Anxiety Inventory; USER-P: Utrecht Scale for Evaluation of Rehabilitation-Participation; CD-RISC: Connor Davidson Resilience Scale; SCSQ: Simplified Coping Style Questionnaire.

**Table 2:** Psychometric tests used.

Test	Main construct	Main Dimensions	N° of items	Scale type	N° Scale Utilizations
CISS (Coping Inventory for Stressful Situations) [38]	Coping strategies	Task-oriented, emotion-oriented and avoidance-oriented coping	48	5-point Likert scale	3
HADS (Hospital Anxiety and Depression Scale) [46]	Anxiety and depression	HADS-A (generalized anxiety symptoms); HADS-D (depressive symptoms);	14	4-point Likert scale	3
AACQ (Assimilative/Accommodative Coping Questionnaire) [38]	Coping strategies	Assimilative and accommodative coping;	16	5-point Likert scale	1
FQCI (Freiburg Questionnaire of Coping with Illness) [47]	Coping strategies	Depressive, active and problem-oriented coping; distraction and self-organization; religious relief and sense-seeking; minimization and wishful thinking	35	5-point Likert scale	1
SF-36 (Short Form Health Survey—36 items) [48]	Quality of life	Physical operation; role limitations due to physical health; body pain; general health; vitality; social functioning; role limitations due to emotional health; mental health	36	Variable Likert scale (up to 6 points per item)	1
QOLIBRI (Quality of Life after Brain Injury) [49]	Quality of life in patients with traumatic brain injury	Cognitive functioning; autonomy in daily life; emotions and feelings; social role and personal relationships; physical status; general well-being	37	5-point Likert scale	1
POMS (Profile of Mood States) [39]	Emotional state	Tension/anxiety; depression/absatisfaction; anger/ostility;vigor/activity; fatigue/inertia; confusion/letting go	65	5-point Likert scale	1
GOSE (Glasgow Outcome Scale Extended) [50]	Disability and recovery after head injury	Death; vegetative state; low severe disability; high severe disability; low moderate disability; moderate high disability; good low recovery; good high recovery	8	8-point Likert scale	1
TICS (Telephone Interview for Cognitive Status) [51]	Cognitive status at a distance by telephone interview	Orientation; memory; attention; language; computational skills; reasoning	11	Binary scale (corrected/incorrect)	1
SPSI-R (Social Problem-Solving Inventory-Revised) [52]	Problem-solving	Positive problem orientation; negative orientation to problems; rational problem-solving style; impulsive/careless problem-solving style; problem-avoidance style	52	5-point Likert scale	1
SS-QOL (Stroke Specific Quality of Life Scale) [53]	Quality of life after stroke	Mobility; family role; social activities; mood; energy; language; self-sufficiency; vision; thinking; personality; higher functioning; activities of daily living	49	5-point Likert scale	1
EQ-5D-5L (EuroQol 5 Dimensions—5 Levels) [54]	Quality of life	Mobility; self-care; daily activities; pain/illness: anxiety/depression;	6	5-point Likert scale; VAS scale (0–100)	1
CES-D (Center for Epidemiological Studies Depression Scale) [55]	Depression	Depressed mood; somatic symptoms; interpersonal relationships; depressive thoughts	20	4-point Likert scale	1
LISAT-9 (Life-Satisfaction Questionnaire-9) [41]	Life satisfaction	General satisfaction with life; satisfaction with personal life management and with friends relationships; satisfaction with: family life, financial situation, leisure time, work, the ability to cope with daily life, sexual life	9	6-point Likert scale	1
SIS 3.0 (Stroke Impact Scale 3.0) [56]	Quality of life and disability	Strengths; hand functionality; activities of daily living; instrumental activities of daily living; mobility; communication; emotion; memory and thinking; participation/role function	59	5-point Likert scale	1
BPI (Brief Pain Inventory) [57]	Pain	Intensity; interference	7	Numeric scale from 0 to 10	1
SCL-90-R (Symptom Checklist-90-Revised) [58]	Psychopathological symptoms	Somatization; obsession–compulsion; interpersonal sensitivity; depression; anxiety; hostility; phobic anxiety; paranoia; psychoticism	90	5-point Likert scale	1
GSE (General Self-Efficacy Scale) [59]	Self-efficacy	Perceived ability to cope with and overcome difficulties, handle daily pressures, and realize personal goals effectively	10	4-point Likert scale	1
COPE (Coping Orientation to Problems Experienced) [42]	Coping strategies	Problem-oriented, emotion-oriented and avoidance-oriented coping; support seeking; acceptance; cognitive restructuring; other specific strategies	60	5-point Likert scale	1
AAQ-II (Acceptance and Action Questionnaire version2) [42]	Psychological flexibility	Acceptance; mindfulness; engagement; behavioral change strategies	10	7-point Likert scale	1
MSPSS (Multidimensional Scale of Perceived Social Support) [60]	Perceived social support	Support from: family, friends, significant persons	12	7-point Likert scale	1
Brief-COPE (Coping Orientation to Problems Experienced Inventory) [45]	Coping strategies	Problem-oriented, emotion-oriented, avoidance-oriented and maladaptive coping; other strategies (practical help request, religiosity, humor)	28	4-point Likert scale	1
FSS (Fatigue Severity Scale) [61]	Severity of fatigue	Fatigue and severity of fatigue; impact of fatigue on certain activities	9	7-point Likert scale	1
MFS (Mental Fatigue Scale) [62]	Mental fatigue	Difficulty in concentrating; cognitive fatigue; Impact on executive and daily life functions	15	3-point Likert scale	1
BDI-II (Beck Depression Inventory—Second Edition) [63]	Depression	Depressed mood; loss of interest or pleasure; sleep alterations; alterations in appetite and weight; feelings of guilt or worthlessness; difficulty concentrating; fatigue or lack of energy; suicidal or self-injurious thoughts	21	4-point Likert scale	1
BAI (Beck’s Anxiety Inventory) [64]	Anxiety	Somatic and cognitive anxiety symptoms	21	4-point Likert scale	1
USER-P (Utrecht Scale for Evaluation of Rehabilitation-Participation) [65]	Social participation	Satisfaction, frequency, restriction of participation	32	Frequency of participation: 6-point Likert; Restriction of participation: Likert at 4; Satisfaction of participation: 5-point Likert	1
CD-RISC (Connor-Davidson resilence scale) [66]	Resilience	Tenacity, strength; optimism	25	5-point Likert scale;	1
SCSQ (Simplified Coping Style Questionnaire) [67]	Coping strategies	Positive and negative coping	20	5-point Likert scale	1
Ad hoc questionnaire [43]	Subjective experiences after subarachnoid hemorrhage	(1) Treatment received (2) Cognitive and physical consequences (3) Coping strategies in daily life (4) Impact on social functioning and daily life (5) Current status: housing, employment, comorbidities	5 open-ended questions	Open-ended questions	1
